# HTRA3 expression in non-pregnant rhesus monkey ovary and endometrium, and at the maternal-fetal interface during early pregnancy

**DOI:** 10.1186/1477-7827-6-22

**Published:** 2008-06-18

**Authors:** Marissa A Bowden, Ying Li, Yi-Xun Liu, Jock K Findlay, Lois A Salamonsen, Guiying Nie

**Affiliations:** 1 Prince Henry's Institute of Medical Research, PO Box 5152, Monash University, Clayton, Victoria 3168, Australia; 2 Department of Obstetrics & Gynaecology, Monash University, Clayton, Victoria 3168, Australia; 3 State Key Laboratory for Reproductive Biology, Institute of Zoology, Chinese Academy of Sciences, Beijing 100080, PR China

## Abstract

**Background:**

HTRA3 is a recently identified member of the mammalian serine protease family HTRA (high temperature requirement factor A). In both the rodent and the human HTRA3 is transcribed into two mRNA species (long and short) through alternative splicing. We have previously shown that HTRA3 is expressed in the mature rat ovary and may be involved in folliculogenesis and luteinisation. HTRA3 is also upregulated during mouse and human placental development. The current study investigated whether HTRA3 is also localised in the primate ovary (rhesus monkey n = 7). In addition, we examined the non-pregnant rhesus monkey endometrium (n = 4) and maternal-fetal interface during early pregnancy (n = 5) to further investigate expression of HTRA3 in primate endometrium and placentation.

**Methods:**

HTRA3 mRNA levels in several rhesus monkey tissues was determined by semiquantitative RT-PCR. Protein expression and localisation of HTRA3 was determined by immunohistochemistry.

**Results:**

Long and short forms of HTRA3 mRNA were detected in the ovary, aorta, bladder, small intestine, skeletal muscle, heart and uterus but not the liver nor the kidney. HTRA3 protein was immunolocalised to the oocyte of all follicular stages in the rhesus monkey ovary. Protein expression in mural and cumulus granulosa cells of late secondary follicles increased significantly compared to granulosa cells of primordial, primary and secondary follicles. Mural and cumulus granulosa cells of antral follicles also showed a significant increase in expression. Staining intensity was higher in the granulosa-lutein cells compared to the theca-lutein cells of corpora lutea (n = 3). In the non-pregnant monkey endometrium, HTRA3 was detected in the glandular epithelium. The basalis endometrial glands showed higher staining intensity than functionalis endometrial glands. During early pregnancy, strong staining for HTRA3 protein was seen in both maternal decidual cells and glands.

**Conclusion:**

We propose that HTRA3 may be involved in folliculogenesis and luteinisation in the primate ovary. Furthermore, similar to previous findings in the human, HTRA3 is possibly a factor involved in and potentially important for primate placentation.

## Background

The high temperature requirement factor A (HtrA) family of serine proteases was first identified in *E. coli *and is conserved from bacteria to humans [1,2]. We identified and cloned a novel gene that is considerably upregulated in the mouse uterus in association with placentation [3]. After cloning the full mRNA sequence of this gene in the human [4] [accession number NM_053044.2, GenBank^® ^Identifier (GI) 24475740] we determined that this protease was structurally related to the previously identified mammalian proteases HTRA1 and 2. Consequently we named this new gene *HTRA3 *[4]. In the human and the rodent, *HTRA3 *gene undergoes alternative splicing (long and short mRNA forms) [3-5]. In both the human and the rodent, the *HTRA3 *gene consists of 10 exons. Long *HTRA3 *is produced by utilizing exons 1 through to 10 except exon 7, whilst the short form is produced by utilizing exons 1 through to 7 [3-5].

The alternative splicing of the *HTRA3 *gene into the long and short form mRNA results in two predicted protein isoforms in both the human and the rodent, 49 kDa and 38 kDa respectively [3,4]. Mammalian HTRA1 and 3 share similar domain organisation including a highly conserved trypsin-like serine protease domain and a C-terminal postsynaptic density protein 95-Discs large-Zona occuldens 1 (PDZ) domain that mediates specific protein-protein interactions. Mammalian HTRA3 also consists of an N-terminal signal peptide, an insulin growth factor binding domain and a kazal-type S protease inhibitor domain [4,6]. Whilst HTRA3 is a serine protease, that fact that it contains an insulin-like growth factor (IGF) binding domain at the N-terminal following the signal peptide, suggests that it can be secreted and may be involved in the IGF system.

In both the human and the rodent, the protein sequence of short HTRA3 is identical to that of the long except that it contains exon 7, whilst long form *HTRA3 *mRNA does not, and it lacks the C-terminal PDZ domain [4,5]. HTRA3 is well conserved between human, mouse and rat with identical bases at 87% and 79% for the mouse and the rat mRNA respectively and 95% similarity for both at the protein level [5,7].

Northern analysis has shown high expression of HTRA3 mRNA in the mouse ovary [3]. In the rat, we have shown a significant increase of long form *HTRA3 *mRNA expression from immature to mature state ovaries [5]. When HTRA3 protein expression was investigated throughout the rat estrous cycle, it was upregulated in the cumulus and mural granulosa cells of late secondary, antral and preovulatory follicles. High levels of HTRA3 protein were also localized in the corpora lutea (CL) [5]. Furthermore long *HTRA3 *mRNA increased significantly with FSH treatment of rat primary granulosa cells in culture for 16 h compared to control, concurrent with luteinisation *in vitro*.

HTRA3 was first identified as a pregnancy related serine protease, uniquely regulated at the time of implantation and placentation in the mouse and human [3,7]. HTRA3 protein has been immunolocalised predominantly to the decidua basalis during mouse implantation [8]. In human endometrium, both glandular and decidual expression of HTRA3 increased significantly in early pregnancy compared to normal endometrium [7]. In particular, decidual expression significantly increased during the first-trimester pregnancy when decidualisation advanced. Furthermore, HTRA3 protein was significantly elevated in the serum of women in first-trimester pregnancy [7]. It has also been reported that there is a clear association between HTRA3 and the human menstrual cycle. The endometrial expression of HTRA3 was found to be highest at the late secretory phase of the cycle, when the endometrium is prepared for maternal-trophoblast interaction should fertilisation occur [7].

In the present study we investigated the expression and regulation of HTRA3 mRNA and protein in the non-pregnant rhesus monkey ovary and endometrium, and at the maternal-fetal interface during early pregnancy. We propose that similar to the findings in the rodent, HTRA3 may be associated with folliculogenesis and luteinisation in the primate ovary. In addition, HTRA3 is expressed in the endometrium during the monkey menstrual cycle and therefore may be associated with early pregnancy in the primate.

## Methods

### Tissue collection and processing

Rhesus monkeys (*Macaca mulatta*) were maintained in the Fu-Zhou Primate Research Centre, China. All experimental work was approved by the Animal Ethics Committee at the Institute of Zoology, Chinese Academy of Sciences. Menstrual cycles of female monkeys were monitored for 2–3 cycles before sampling (n = 10). One ovary and uterine tissue were collected 3, 2 and 1 day before (ov-3, ov-2 and ov-1 respectively), and 5, 9, 10, 11, 12, 14 and 15 days after (ov+5, ov+9, ov+10, ov+11, ov+12, ov+14 and ov+15 respectively) ovulation. For pregnant monkeys (n = 5), animals were permitted to mate over a period of 3 days at the anticipated time of ovulation, the second day of mating was designated day 0 of pregnancy. The presence of a conceptus was confirmed by ultrasound diagnosis and uterine tissue collected. At the appropriate time of either the cycle (ov-3 to ov+15) or pregnancy (d22 or d35), the monkeys were sacrificed humanely, the ovaries, uterus, aorta, bladder, small intestine, skeletal muscle, heart, liver and kidney were removed. Appropriately selected wedges of the ovaries and the uterus were fixed in buffered formalin overnight at 4°C, washed in Tris-buffered saline (pH 7.4) (TBS) and processed to paraffin wax blocks. The remaining sections of the ovary and the uterus and all other tissue types were collected in RNA later (Ambion, CA, US) and stored at -80°C for RNA extraction.

### RNA extraction, purification and quantitation

RNA was extracted from adult monkey tissue by homogenisation in Trizol reagent (Qiagen Sciences, MD) according to the manufacturer's instructions. All samples were treated with RNase-free DNase (Ambion) and the concentration and purity assessed by spectrophotometry and agarose gel electrophoresis.

### Reverse transcription and traditional PCR

**Figure 1:**
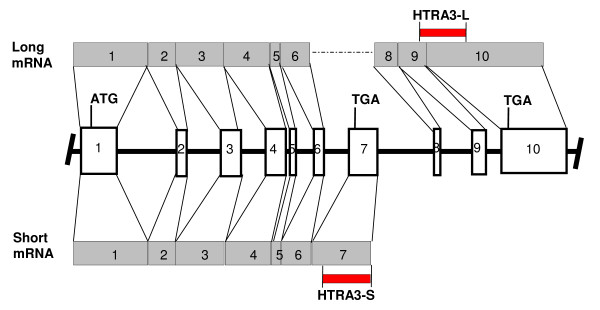
**Alternative splicing of the HTRA3 gene and strategy to analyze the two transcripts by RT-PCR**. A schematic illustration of the alternative splicing of the HTRA3 gene to show how the long and the short transcripts are generated and analyzed. ATG, start codon; TGA, stop codon. Fragment HTRA3-L, amplified by *HTRA3-L *F (forward) and *HTRA3-L *R (reverse) primers listed in Table 1, across exon 9 and 10, represents the long specific transcript; fragment HTRA3-S, amplified by *HTRA3-S *F (forward) and *HTRA3-S *R (reverse) primers listed in Table 1, located on exon 7, represents the short specific transcript.

Total RNA (1 ug) was reverse transcribed as previously described [9]. A 2 μl aliquot of RT product was amplified in a total volume of 25 μl using 2 × PCR-Master mix with added magnesium (Promega) and 10 pmol sense and antisense primers for long and short form *HTRA3 *and *18S *rRNA as outlined in Table 1. Primers for long form *HTRA3 *were designed to specifically amplify exons 9 and 10, therefore amplifying exons specific to the long form and a region that spans an intron (Fig 1). Primers for short form *HTRA3 *were designed to amplify a region of exon 7, an exon specific to the short form HTRA3 (Fig 1). PCR was performed using a conventional PCR block cycler (Hybaid PCR Express) with an annealing temperature of 58°C or 53°C for 30 cycles for the long form and the short form of *HTRA3 *respectively. Cycle numbers in the linear phase were used and *18S *(with an annealing temperature of 58°C for 20 cycles) was used as a control. PCR products were analysed by electrophoresis on a 1% (w/v) agarose/TBE gel and bands visualised via ethidium bromide staining. The bands were excised and the identity of each PCR product was also verified by DNA sequencing in initial experiments.

**Table 1:** Primer sequences used for block RT-PCR analysis of *HTRA3*

Primer		Sequence (5' to 3')
*HTRA3-L*	F	ATG CGG ACG ATC ACA CCA AG
	R	CGC TGC CCT CCG TTG TCT G

*HTRA3-S*	F	GAG GGC TGG TCA CAT GAA GA
	R	GCT CCG CTA ATT TCC AGT

*18S*	F	CGG CTA CCA CAT CCA AGG AA
	R	GCT GGA ATT ACC GCG GCT

*HTRA3-L*, long form HTRA3 mRNA; *HTRA3-S*, short form HTRA3 mRNA.

F, forward primer; R, reverse primer

### Immunohistochemistry

Five-micron serial sections of ovarian and uterine tissue were subjected to standard immunohistochemistry. All ovarian sections underwent immunostaining in a single experiment as did all uterine sections. For each section of tissue, a serial tissue section was used as a negative control. Following antigen retrieval by microwaving the sections for 5 min in 0.1 M citric acid buffer (pH 6.0), the endogenous hydrogen peroxidase activity was quenched by incubating the sections with 3% H_2_O_2_. For the ovarian tissue, non-specific binding was blocked by pre-incubation with a blocking buffer containing high salt TBS (0.3 M NaCl in 50 mM Tris, pH 7.6), 0.1% Tween 20, 15% rabbit serum and 2% normal human serum for 1 h at room temperature. A similar blocking buffer and method was used for the uterine tissue except 12% rabbit serum and 6% fetal calf serum were used in the buffer. The primary antibody [sheep anti-HTRA3 antibody #153 [7] (2 μg/ml for ovarian tissue, 0.5 μg/ml for uterine tissue) or pre-immune sheep IgG as a negative control, at the same concentration as the primary antibody] was incubated in a blocking buffer containing high salt TBS, 0.1% Tween 20, 10% rabbit serum and 2% normal human serum at 37°C for 1 h and washed with high salt TBS plus 0.6% Tween. The secondary antibody [#BA-6000, biotinylated rabbit anti-sheep IgG (1:200, Vector Laboratories, Burlingame, CA)] was applied in the same blocking buffer as the primary antibody for 30 min at room temperature. Positive immunostaining was revealed by incubating the sections with an avidin-biotin-complex conjugated to horseradish peroxidase (DakoCytomation, Botany, Australia) in the dark for 30 min at room temperature, followed by the application of the peroxidase substrate 3,3'-diaminobenzidine (DakoCytomation) leading to a brown precipitate. The sections were counterstained with Harris hematoxylin. Microscopy was performed using an Olympus BH2 microscope fitted with a Fujix HC-2000 high resolution digital camera (Fujix, Tokyo, Japan).

To determine the expression level and cellular localization of HTRA3 protein in the non-pregnant ovary, ovaries collected at ov-3, ov-2, ov+5, ov+9, ov+11, ov+12 and ov+14 were examined (n = 1 monkey for each day). Follicles were counted and investigated from all 7 monkeys (primordial follicles, n = 158; primary follicles, n = 119; secondary follicles, n = 78; late secondary follicles, n = 4; antral follicles, n = 5 (antral follicles were observed in monkeys at ov-3 and ov-2), corpora lutea, n = 3 (CLs were observed in monkeys at ov+11, ov+12 and ov+14). Non-pregnant uterine tissues sampled at ov-1 (n = 3), ov+5 (n = 3), ov+10 (n = 3) and ov+15 (n = 2) of the menstrual cycle were also examined. The relative intensity of the immunostaining was scored semiquantitatively by two independent observers; zero representing no staining and four representing maximal staining. Such analysis of immunostaining has previously been reported [10,11]. The scores for each type of cellular subtype in each different follicle type investigated by each observer were then averaged. Comparisons of staining intensities between cellular subtypes within different follicle types were performed using One-way ANOVA, followed by Tukey's *post hoc *test using PRISM v4 for Windows (GraphPad Software Inc, San Diego, CA). The staining intensity of the endometrial glandular epithelium of non-pregnant monkeys at different stages of the cycle was scored and analysed in a similar fashion, with the quantitation resulting in the average of the signal. P < 0.05 was considered statistically significant.

The expression of HtrA3 at the maternal-fetal interface was determined by examining implantation sites (including the uterus and the placenta) from d22-d35 pregnant monkeys (n = 5). For these tissues, trophoblast cells were identified by immunostaining for cytokeratin using a mouse anti-human cytokeratin monoclonal antibody (7 μg/ml, #34902, BD Biosciences, San Jose, CA) as the primary and a biotinylated horse anti-mouse IgG (1:200, #BA-200, Vector Laboratories) as the secondary antibody, following antigen retrieval as for immunostaining of HtrA3.

## Results

### Expression of HTRA3 mRNA in adult rhesus monkey tissue

**Figure 2:**
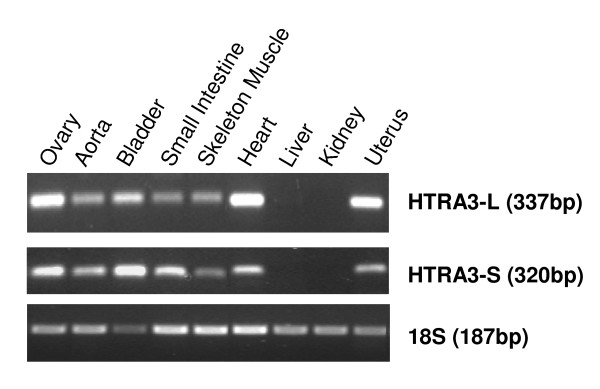
***HTRA3 *mRNA expression in various adult rhesus monkey tissues**. Amplification of long *HTRA3 *(HTRA3-L), short *HTRA3 *(HTRA3-S) mRNA and *18S *rRNA in rhesus monkey ovary, aorta, bladder, small intestine, skeletal muscle, heart, liver, kidney and uterus. The PCR product size (bp) for each transcript is indicated.

Using RT-PCR, the long and short forms of *HTRA3 *mRNA were detected in the ovary, aorta, bladder, small intestine, skeletal muscle, heart and uterus but not the liver nor the kidney (Fig 2). The long form *HTRA3 *mRNA product size was 337 base pairs (bp) while the short form *HTRA3 *mRNA was 320 base pairs. The highest level of the long form *HTRA3 *mRNA was in the ovary, heart and uterus (Fig 2). The highest expression of short form *HTRA3 *mRNA was in the ovary and bladder (Fig 2). *18S *was detected in all adult rhesus monkey tissues investigated (Fig 2). The RNA extracted from ovaries at each stage of the cycle was analysed by traditional RT-PCR, however there was no difference in the expression of either the long form *HTRA3 *or the short form *HTRA3 *between the different stages (data not shown).

### Localization of HTRA3 protein in the ovary of non-pregnant rhesus monkey

**Figure 3:**
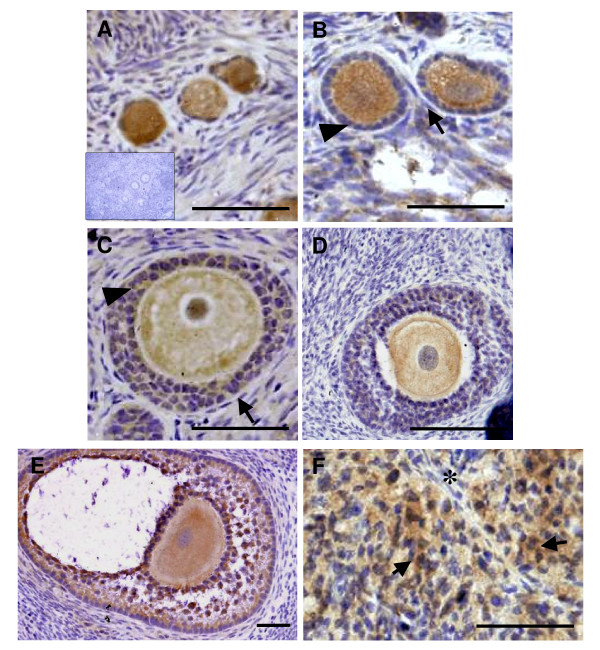
**HTRA3 protein immunolocalisation in the adult rhesus monkey ovary**. Representative photomicrographs of HTRA3 immunolocalisation in follicular sub-populations in adult rhesus monkey ovary. A, primordial follicles with insert of negative control; B, primary follicles with granulosa cells highlighted by the arrowhead and newly forming thecal cells by the arrow; C, secondary follicle, granulosa cells highlighted by arrowhead and thecal cells by arrow; D, late seondary follicle; E, antral follicle; F, granulosa-lutein cells (arrow) and theca-lutein cells marked by asterisk (*) of newly forming CL. Scale bar = 50 μm

**Figure 4:**
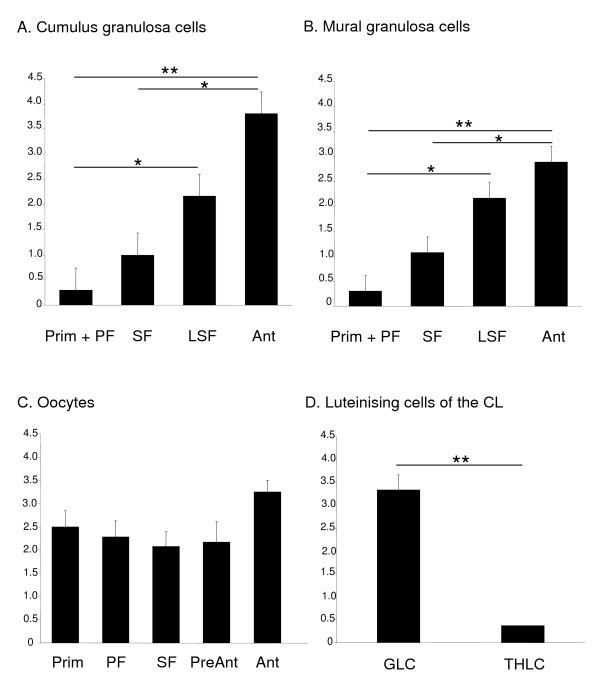
**Intensity of staining for HTRA3 protein in the adult rhesus monkey ovary**. Mean density (± SEM) of HTRA3 immunostaining in the monkey ovary. A, cumulus granulosa cells; B, mural granulosa cells; C, oocytes; D, luteinising cells of the CL. Prim, primordial follicle (n = 158); PF, primary follicle (n = 119) (Prim + PF, n = 277); SF, secondary follicle (n = 78); LSF, late secondary follicle (n = 4); Ant, antral follicle (n = 5); GLC, granulosa-lutein cells; THLC, theca-lutein cells (n = 3 CL). ** P < 0.01, *P < 0.05.

Primordial follicles (Fig 3A) showed staining in the oocyte, however the squamous granulosa cells surrounding the oocyte were negative for HTRA3 protein. A similar pattern was seen in the primary follicles (Fig 3B arrowhead). The newly forming thecal cells surrounding the granulosa cells were negative for HTRA3 (Fig 3B arrow). Expression of HTRA3 protein began to increase in the secondary follicles with the granulosa cells (Fig 3C arrowhead) showing moderate staining for HTRA3 whilst the thecal cells showed no staining (Fig 3C arrow). When the intensity of staining in the granulosa cells was scored, there was no significant difference between primordial and primary and secondary follicles (Fig 4A and 4B). HTRA3 protein expression in the granulosa cells significantly increased (P < 0.05) from primordial and primary follicles to the cumulus and mural granulosa cells of the late secondary follicles (Fig 3D and Fig 4A and 4B). The thecal cells remained negative. A significant increase in the intensity of granulosa cell staining for HTRA3 protein was observed in the antral follicles (Fig 3E and 4A and 4B). HTRA3 protein in both the cumulus and the mural granulosa cells was significantly higher in antral follicles than in primordial and primary follicles (P < 0.01), and secondary follicles (P < 0.05) (Fig 4A and 4B). The cumulus granulosa cells in the antral follicles (Fig 4A) showed more intense staining for HTRA3 compared to the mural granulosa cells (Fig 4B). The thecal cells of antral follicles were negative for HtrA3 protein. In the CL the granulosa-lutein cells (Fig 3F arrows) showed significantly (P < 0.01) higher expression of HTRA3 protein than the theca-lutein cells (Fig 3F asterisk and 4D).

High expression of HTRA3 protein was observed in the oocytes of all follicles. This level of expression was not significantly different between follicle types (Fig 3A-E and Fig 4C).

### Localization of HTRA3 protein in the endometrium of non-pregnant/non-mated rhesus monkey

**Figure 5:**
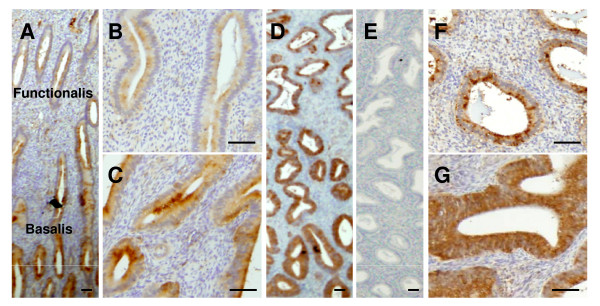
**HTRA3 protein immunolocalisation in the endometrium**. A, ov+5, the side of the basalis glands is presented on the bottom of the image. B-C, functionalis (B) and basalis (C) glands of (A) at a higher magnification. D, ov+10, tissue orientation same as A. E, negative control whereby pre-immune sheep IgG was used as the primary antibody on an adjacent section of D. F-G, functionalis (F) and basalis (G) glands shown of (D) at a higher magnification.

**Figure 6:**
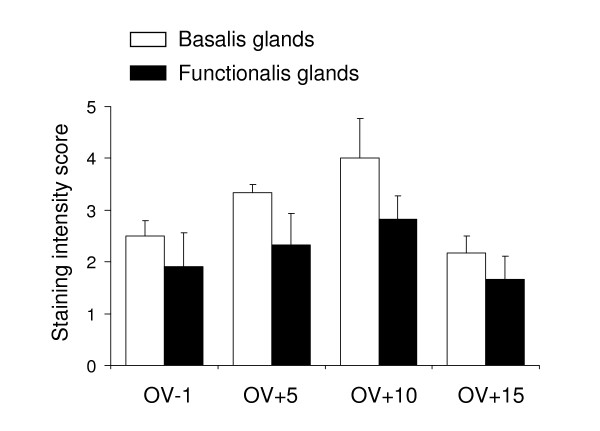
**Intensity of staining for HTRA3 protein in the endometrial glands of non-pregnant, non-mated rhesus monkeys**. Mean density (± SEM) of HtrA3 immunostaining in endometrial glands (basalis and functionalis respectively). ov, ovulation; ov-1, one day before ov; ov+5, ov+10 and ov+15, 5, 10 and 15 days after ov respectively. n = 3 for all stages except for ov+15 (n = 2).

In the non-pregnant monkey endometrium, HTRA3 was detected primarily in the glandular epithelium at all examined stages of the cycle (Fig 5A–G,). Within each sample, however, not all glands were stained with equal intensity, rather zonal variations in staining were observed. In general, glands close to the myometrium were stained stronger than those close to the uterine lumen (Fig 5A and 5D). To take this difference within each sample into consideration when comparing different samples, we arbitrarily divided the glands on each section into two groups (outer and inner) according to their location and scored separately. The outer glands were defined as those located in the upper two-thirds of the endometrium near the lumen known as the functionalis and the inner glands in the lower third of the endometrium near the myometrium known as the basalis. In all sections examined, the basalis glands on average, stained more strongly than the functionalis glands (Fig 5B and 5C, F and 5G, and Fig 6). When different stages of the cycle were compared, the two groups of glands showed a very similar trend of immunostaining: lower intensity at ov-1 (Fig 5B–C, Fig 6), higher at ov+5 and maximal at ov+10 (Fig 5F–G, Fig 6), but then decreasing again at ov+15 (Fig 6). Statistical analysis, however, did not reveal these differences to be significant.

### Localization of HTRA3 at rhesus monkey implantation sites

**Figure 7:**
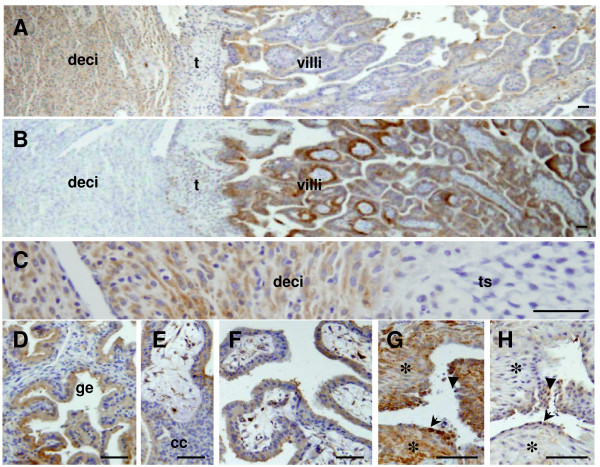
**HTRA3 immunolocalisation at the maternal-fetal interface**. A, a low power view of an implantation site. C, a higher power view of A showing the decidual-trophoblast shell junction. D, maternal glands. E, anchoring villi, F, floating villi. Cytokeratin staining in implantation sites is shown in panels B and G. B, an adjacent section of A stained for cytokeratin; G, extravillous endovascular trohoblasts (EVTs) surrounding a blood vessel stained for cytokeratin; EVTs outside the vessel are highlighted by asterisks (*), and those lining and inside the vessel by the arrow and arrowhead respectively. H, an adjacent section of G stained for HTRA3. Deci, decidual cells; t, trophoblast cells; ts, trophoblast shell; ge, glandular epithelium; cc, trophoblast cell column. Scale bar = 50 μm.

The earliest implantation site available was on day 22 and the latest on day 35 of pregnancy. HTRA3 staining pattern was the same between these days and an implantation site on day 35 is shown (Fig 7A). Both maternal decidual cells (Fig 7A and 7C) and glands (Fig 7D) were strongly stained for HtrA3 during pregnancy. In contrast, trophoblast cells, which were positively identified by their staining for cytokeratin (Fig 7B), showed weak staining for HTRA3 depending on the subtypes (Fig 7A). Trophoblast shell was completely negative for HTRA3 (Fig 7C), so were most of the cells in the trophoblast cell columns in the anchoring villi (Fig 7E). On the other hand, the syncytial trophoblast on the exterior of the anchoring (Fig 7E) and the floating (Fig 7F) villi was positive. Some but not all villous cytotrophoblast was positively stained for HTRA3, the intensities were generally weaker than in the adjacent syncytiotrophoblasts (Fig 7F).

A few blood vessels in the decidua close to the trophoblast shell were surrounded by a thick rim of large number of extravillous cytotrophoblasts (EVTs) which stained strongly for cytokeratin (Fig 7G, asterisks). The wall of such blood vessels could no longer be clearly defined, and it was apparent that some trophoblast cells had replaced the endothelium (Fig 7G, arrow) and some were inside the vessel (Fig 7G, arrowhead). When serial sections were immunostained for HTRA3, the EVTs outside the blood vessel were negative (Fig 7H, asterisk), whereas those lining or inside the vessel (Fig 7H, arrow and arrowhead respectively) were weakly positive. Except those EVTs that were closely associated with blood vessels, no interstitial EVTs distributed away from blood vessels was identified on our sections.

## Discussion

This study confirmed a correlation between firstly, HTRA3 during folliculogenesis and luteinisation in the rhesus monkey ovary, and secondly between endometrial function and the maternal-fetal interface during early pregnancy in the rhesus monkey.

A significant increase in HTRA3 protein expression from primordial and primary follicles through to antral follicles was observed in granulosa cells with the exception of theca cells. This increase in HTRA3, particularly in the granulosa cells, with the maturation of follicles is similar to that seen in the rat [5] and indicates that HTRA3 is associated in the process of folliculogenesis not only in rodents, but also in primates.

Both the cumulus cells surrounding the oocyte and the mural granulosa cells lining the follicle wall of late secondary and antral follicles showed significant upregulation of HTRA3, however the cumulus granulosa cells stained more strongly for the protein. An important stage in the maturation of follicles is the division of granulosa cells into cumulus and mural cells. Several factors are involved in this differentiation step including members of the transforming growth factor-β (TGF-β) superfamily. In the mouse, lack of oocyte derived bone morphogenetic protein-15 (BMP-15) results in defective cumulus cell differentiation and cumulus expansion [12,13]. Follicles fail to progress beyond the late secondary stage in the absence of oocyte derived growth differentiation factor-9 (GDF-9) [14,15]. Consequently it is clear that the cumulus cells are involved in promoting oocyte growth and developmental competence through bidirectional interactions with the oocyte [13,16]. HTRA3 is a newly identified protein [3,4] and its function is still unclear. Supporting a possible role for HTRA3 protease action in the differentiation of granulosa cells into cumulus and mural cells is the report that HTRA3 can bind to various TGF-β superfamily members and can inhibit the signalling of BMP-4, BMP-2 and TGF-β 1 [17]and that HTRA3 protein was upregulated in the cumulus granulosa cells and the oocyte of antral follicles in the rat ovary [5] and in the primate in the current study.

HTRA3 may also be involved in the formation of a local extracellular matrix (ECM) by the cumulus cells which envelopes the cumulus oocyte complex (COC). This process plays a vital role in ovulation [18,19]. HTRA3 exhibits substrate specificity toward β-casein and certain ECM proteoglycans [17] implying that HTRA3 may directly modulate the ECM microenvironment of the COC to facilitate ovulation.

High expression of HTRA3 was seen in the granulosa-lutein cells of the CL implying a role for this serine protease in luteinisation. This is supported by the localisation of HTRA3 in the rat CL [5]. A large number of proteinases involved in folliculogenesis and luteinisation are found in the CL. Proteoglycans such as decorin have been found in bovine CLs [20]. Decorin is a well known modulator of the ECM and recombinant HTRA3 can degrade ECM proteins such as decorin [17,21]. This suggests a role for ovarian derived HTRA3 in the regulation of the ECM microenvironment of the CL for the process of luteinisation.

In addition, oocytes in all follicles stained positive for HTRA3 protein. In most species, including humans, oocyte health, follicle growth and ovulation are dependent on some level of oocyte derived TGF-β superfamily members [22,23]. As HTRA3 is reported to be able to bind to a broad range of TGF-β superfamily members and inhibit TGF-β signalling [17,24], and the present study has shown that HTRA3 is expressed in similar cells in the ovary as TGF-β superfamily members (for a review see [25]), it is possible that HTRA3is involved in the maintenance of the health of oocytes.

During the rhesus monkey menstrual cycle, HTRA3 was localised primarily in the endometrial glands. Interestingly expression of HTRA3 protein in these glands increased 5 days following ovulation and was maximal 10 days after ovulation. During this secretory phase of the cycle in the primate, the endometrium prepares for maternal-trophoblast interaction should fertilisation occur. Following implantation, HTRA3 was expressed strongly in the glands and decidual cells.

The high expression of HTRA3 protein in both the maternal decidual cells and the glands during early pregnancy correlates with previous findings in the human. HTRA3 was in particular significantly upregulated during first-trimester pregnancy when decidualisation is fully advanced and is also increased as stromal cells decidualise *in vitro *[7]. Many growth factors and ECM proteins produced by the decidua regulate trophoblast proliferation, differentiation and invasion. It has been shown that HTRA3 and HTRA1 are able to degrade a number of ECM proteins [17,21]. It is therefore possible that HTRA3 is directly involved in the decidua ECM microenvironment, assisting trophoblast invasion. In addition, strong expression of TGF-β superfamily members has been reported in the decidua [26] and these decidual TGF-βs have been demonstrated to be able to inhibit trophoblast invasion via paracrine manners [26]. This suggests that in the decidua, HTRA3 may inhibit TGF-β signalling, thereby regulating trophoblast migration and invasion.

During early pregnancy, HTRA3 expression was also detected in synctiotrophoblasts and endovascular EVTs in the rhesus monkey. This is consistent with studies reported in the human [7]. However, expression of HTRA3 in the trophoblast shell was below detection in the rhesus monkey in contrast to that seen in the human [7]. This may highlight the variation between the rhesus monkey and the human.

Both the rodent and the human HTRA3 and HTRA1 protein sequences include an IGF binding domain at the N-terminus [3,27] suggesting that these serine proteases may be involved in the IGF/IGFBP system. Indeed, it has been reported that HTRA1 cleaves IGFBP-5 and that the inhibition of its protease activity suppresses IGF-I action [28]. The IGF/IGFBP system is vital in modulating the maternal-fetal interface for the establishment and maintenance of pregnancy and all components of the system are expressed in the endometrium/decidua or placenta at various stages of pregnancy in all species [29-31]. An important factor in the co-ordination of the IGF/IGFBP system is proteolysis of IGFBPs leading to increased IGF bioactivity [32,33]. It is thus possible that HTRA3 may be capable of modulating the IGF/IGFBP system particularly during placentation. Further studies are required to elucidate the precise molecular mechanisms of HTRA3 during this process.

## Conclusion

This study has demonstrated a clear association between HTRA3 expression and folliculogenesis and luteinisation in the rhesus monkey ovary. In addition we have reported a dynamic correlation between HTRA3 and endometrial function at the maternal-fetal interface during early pregnancy in the rhesus monkey supporting previous findings in the human [7]. These results suggest a role for HTRA3 in primate female reproduction. In order to determine the mechanisms of HTRA3 in female reproduction, the next vital step in research is to determine HTRA3 substrate specificity.

## Competing interests

The authors declare that they have no competing interests.

## Authors' contributions

All authors have read and approved the manuscript. MAB performed immunohistochemistry on the ovary tissue, analysed the data, performed the statistical analysis and drafted the manuscript, Y-X L collected the rhesus monkey tissue, YL performed semiquatitative RT-PCR and immunohistochemistry on the endometrium and maternal- fetal interface tissue, JKF and LAS participated in the design of the studies, GN designed the studies.
